# Case Report: Re-Treatment With Lu-DOTATATE in Neuroendocrine Tumors

**DOI:** 10.3389/fendo.2021.676973

**Published:** 2021-04-15

**Authors:** Elena María Vida Navas, Alberto Martínez Lorca, Aintzane Sancho Gutiérrez, Lucia Sanz Gómez, Teresa Navarro Martínez, Enrique Grande Pulido, Alfredo Carrato Mena, Pablo Gajate Borau

**Affiliations:** ^1^ Medical Oncology Department, Hospital Universitario Ramón y Cajal, Instituto Ramón y Cajal de Investigación Sanitaria, Madrid, Spain; ^2^ Nuclear Medicine Department, Hospital Universitario Ramón y Cajal, Madrid, Spain; ^3^ Medical Oncology Department, Hospital Universitario Cruces, Baracaldo, Spain; ^4^ Medical Oncology Department, MD Anderson Cancer Center Madrid, Madrid, Spain

**Keywords:** neuroendocrine tumors, Lu-DOTATATE, neuroendocrine neoplasms, peptide receptor radionuclide therapy, case report

## Abstract

Peptide receptor radionuclide therapy (PRRT) is an established treatment in advanced neuroendocrine tumors (NETs), which overexpressed somatostatin receptors. However, after progression there are a limited number of available treatments. We want to share a case report about a patient with a NET re-treated with ^177^Lu-DOTATATE and a literature review about salvage treatment with PRRT. We present a 26-year-old man who started with pelvic pain and after a biopsy of a retro-rectal mass observed in a magnetic resonance was diagnosed with an advanced neuroendocrine tumour. After progression to lanreotide, everolimus and sunitinib, treatment with ^177^Lu-DOTATATE was initiated, achieving an excellent response with a progression free survival (PFS) of 38 months. At the time of progression, re-treatment with ^177^Lu-DOTATATE was decided, showing a new partial response, which is currently stable after 15 months. The patient had not presented significant treatment-related toxicity. Although there are no randomized phase III trials or a consensus about the number or dose of cycles, there is evidence about the efficacy and low toxicity of salvage treatment with ^177^Lu-DOTATATE in NETs. Median progression-free survival ranges from 6 to 22 months. Toxicity is mostly hematologic (anemia and neutropenia), 4-7% grade 3/4.

## Case Report: Re-treatment With Lu-DOTATATE in Neuroendocrine Tumors

## Introduction

Neuroendocrine neoplasms (NENs) are a heterogeneous group of neoplasms, which arise in neuroendocrine cells of the mucous membranes with an incidence of 6-7 cases per 100,000 people in the United States, with an increase in the last years (1). NENs can originate from different organs, although most do so from the lungs, the pancreas, and the gastrointestinal tract. 

Based on their histological differentiation and grade that correlate with the proliferation index ki67 and mitotic rate, NENs can be classified in well-differentiated neuroendocrine tumors (NETs) and poorly-differentiated neuroendocrine carcinomas (NECs), which differ in their treatment because of their more aggressive behavior (2). NETs can be divided in low-grade (grade 1) and intermediate grade (grade 2). In addition, there is a subset of NENs that appear histologically well-differentiated with a high proliferation rate. The 2019 WHO classification of NENs recognizes a category of well-differentiated NETs with high-grade (grade 3) (3). Furthermore, NETs can be classified based on their clinical characteristics in functional or nonfunctional tumors, depending on its capacity of secreting hormones, such as serotonin, insulin, gastrin, or glucagon. 

The majority of NETs overexpresses somatostatin receptors that are used as a diagnostic and therapeutic target (4). Somatostatin analogs (octreotide and lanreotide) are standard first line of treatment in the advanced disease (5–7). In the last decades there has been an improvement in the knowledge of molecular biology of NETs, and many clinical studies have been launched with targeted therapies involved in tumorigenesis, such as mammalian target of rapamycin (mTOR) inhibitors or tyrosine kinase inhibitors (TKIs), achieving the approval of everolimus and sunitinib (sunitinib only in pancreatic NET) (8–14). Although there are several therapeutic options, limited response rates and significant toxicities make new approaches necessary.

In this context, peptide receptor radionuclide therapy (PRRT) arose as a new targeted option against NETs, delivering radionuclides directly to tumour cells (15). First clinical studies analyzed the efficacy of somatostatin analogs labeled to radionuclides of Yttrium or Indium, but there was an important hematologic and renal toxicity (16, 17). Lately, Lutetium-177(^177^Lu)-DOTATATE has shown its efficacy with a better safety profile, and it has been established as a valid option in metastatic NETs treatment, with data of clinically relevant long responses. In this context, NETTER-1 is a phase III clinical trial that assessed the activity of ^177^Lu-DOTATATE compared to high dose of octreotide in patients with advanced midgut NETs (15). ^177^Lu-DOTATATE increased the objective response rate (ORR) (18% vs 3%; p < 0.001) and the progression free survival (PFS) (28.4 vs. 8.5 months; HR 0.21, 95% confidence interval (CI) 0.14–0.33, p < 0.0001). Although data is still immature, it showed a trend toward improved overall survival (OS) (median not achieved vs. 27.4 months, HR 0.46, 95% CI 0.14–1.5). European centers of reference have published large series of patients with gastroenteropancreatic and bronchial NETs treated with PRRT (18, 19). These series confirmed the benefit of ^177^Lu-DOTATATE in NETs of primary tumour sites other than the midgut.

Despite the benefit of systemic treatment in NETs the majority of patients recurs and need a new therapeutic alternative. In this way there is an increasing interest of salvage PRRT with ^177^Lu-DOTATATE in patients with NETs. Here, we present a patient with a NET treated with salvage ^177^Lu-DOTATATE and review the literature of salvage PRRT in NETs.

## Clinical Case

An 18-year-old Caucasian man without any relevant medical history, surgeries, or medical family history, was evaluated for intense pelvic pain of several months of evolution, refractory to common analgesics. He did not report diarrhea or other symptoms suggesting carcinoid syndrome. Physical examination, including abdomen and pelvis, did not show significant findings. The initial laboratory test did not present biochemical or hematological abnormalities. Due to the lack of clinical or analytical findings and the severity of the pelvic pain, a magnetic resonance was performed, showing a 4,3 x 3,3 x 4 cm retro-rectal mass, and the biopsy revealed a low-grade neuroendocrine tumour.

The patient underwent surgery in June 2012. However, the complete resection was not feasible due to sacrum infiltration. The histology confirmed a neuroendocrine tumour grade 2 and a proliferation index Kinett 67 of 15%, with nodal invasion, pT4pN1Mx (stage IIIB AJCC 8^a^ ed.). Post-surgical computerized tomography (CT) demonstrated tumour persistence. A second surgery was performed in September 2012 achieving a complete resection, followed by adjuvant radiotherapy in December 2012.

In June 2013, a CT scan showed multiple bone metastases. A somatostatin receptor scintigraphy (SRS) demonstrated radiotracer uptake in the occipital bone and the third lumbar vertebrae. Treatment with lanreotide, 120 mg every four weeks, was initiated. Nevertheless, in November 2013 the patient presented new bone progression. 

Second line treatment with everolimus, 10 mg daily, was started. Despite initial benefit, in September 2014 a new CT scan and a bone scintigraphy revealed an increase in the number of bone metastases. At that time, sunitinib, 37,5 mg daily, was initiated with stable disease in radiological assessments. However, after two years of treatment progressive disease was observed. In January 2016, the CT scan demonstrated new bone metastases located in the mandible and femur.

**Figure 1:**
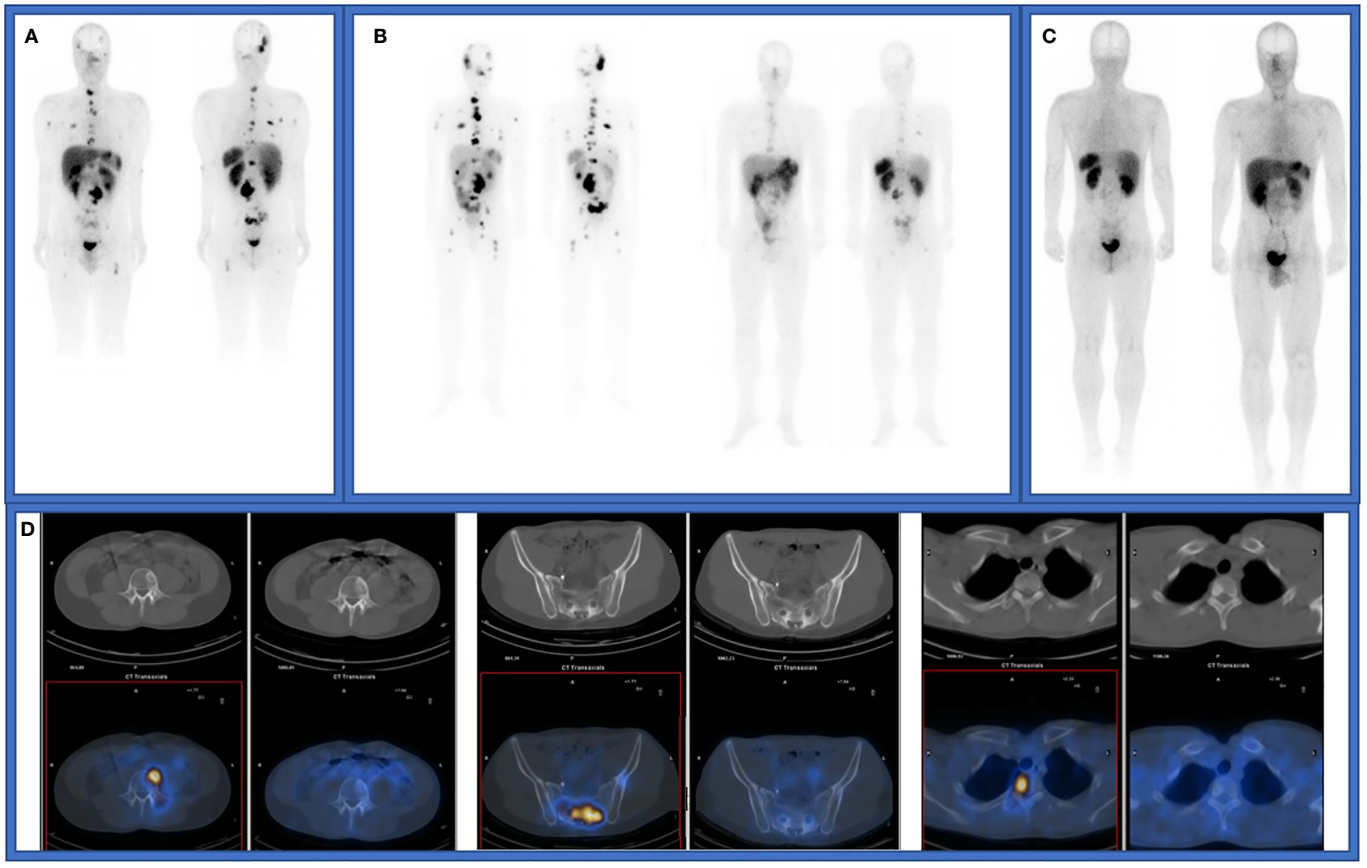
**(A)** Octreoscan imaging in December 2015, before de first treatment with 177Lu-DOTATATE. **(B)** SPECT-CT studies after first and fourth 177Lu-DOTATATE doses. **(C)** Octreoscan imaging in November 2016, after finishing first treatment with 177Lu-DOTATATE. **(D)** SPECT-CT studies in December 2015 and November 2016.

In February 2016, the patient was referred to our center and ^177^Lu-DOTATATE treatment was offered with four doses (7,4 GBq (200 mCi) every eight weeks). The first dose was administered in April 2016. The SPECT-CT, performed after the second dose, demonstrated a decreased number of bone metastases with a lower radiotracer uptake. The treatment was completed in September 2016 with an important clinical and radiological benefit observed in the SRS (**Figure 1**) and in the CT scan by RECIST 1.1. No adverse events related to ^177^Lu-DOTATATE were observed.

**Figure 2:**
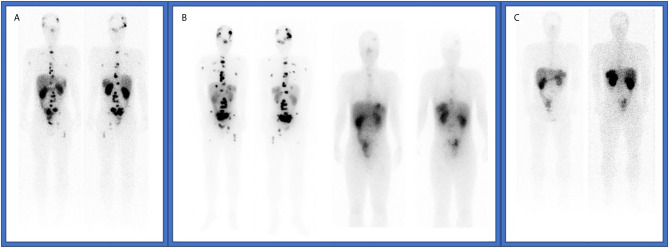
**(A)** Octreoscan imaging at tumor recurrence in June 2019. **(B)** SPECT-CT studies after fifth and sixth 177Lu-DOTATATE doses. **(C)** Octreoscan imaging after complete 177Lu-DOTATATE re-treatment.

**Figure 3:**
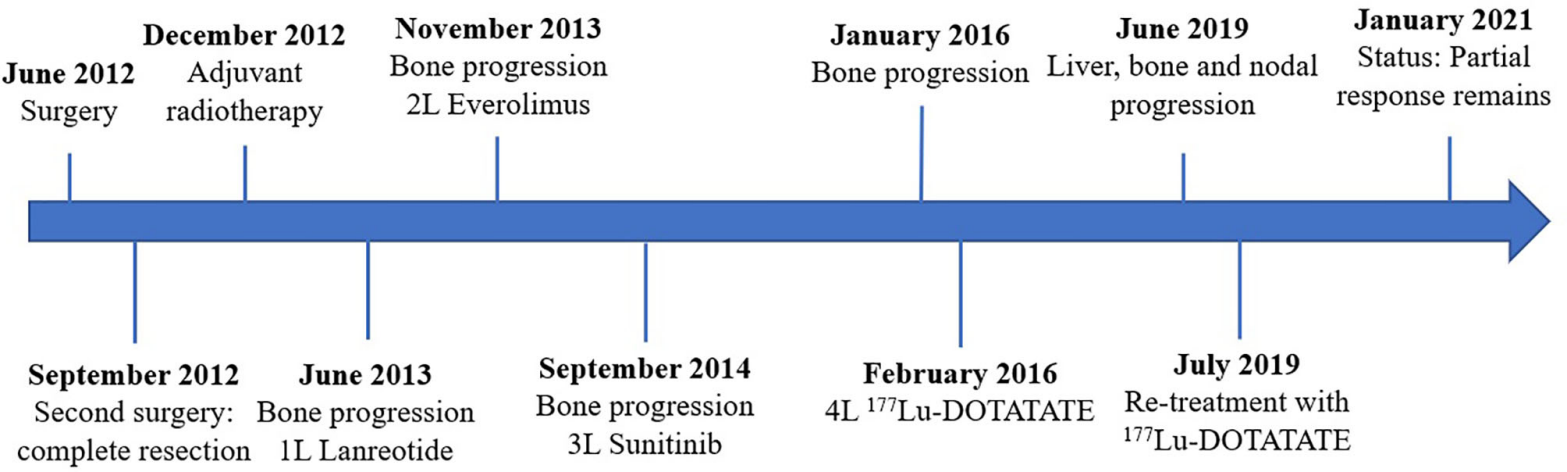
Historic evolution of the patient.

The patient started follow-up with physical examination, laboratory tests, CT scan and SRS every three months, and the disease was controlled until June 2019, when a CT scan showed liver, bone and nodal progression (**Figure 2**). Due to the lack of valid alternative therapeutic options and the excellent previous response, it was decided in a multidisciplinary committee to re-treat with two more doses of 7,29 GBq (197 mCi) of ^177^Lu-DOTATATE every eight weeks. As in the first treatment, the dose received was assessed by dosimetry after each administration. The treatment was administered in July and September 2019, achieving again a new response observed in the CT scan by RECIST 1.1 and in the SRS, without clinically relevant hematologic or renal toxicity, and started again follow up. In the current moment, the patient maintains partial response achieved with ^177^Lu-DOTATATE (**Figure 3**).

## Discussion

This case report is a good example of the efficacy and safety of salvage therapy with ^177^Lu-DOTATATE in heavily pretreated patients after an initial response to PRRT, an especially challenging context with a limited number of alternatives.

Currently, ^177^Lu-DOTATATE is an established therapeutic option for the treatment of metastatic NETs. However, there is a lack of evidence for salvage therapy. Recently, published studies have shown the efficacy and acceptable tolerance of re-treatment with PRRT (**Table 1**). Nevertheless, these studies are heterogeneous and mostly retrospective, with significant differences between them regarding the patients included, the cumulative dose of PRRT, or the radiolabeled drug used.

**Table 1:** Published studies that evaluate salvage with PRRT.

Study (year of publication)	Number of patients (n)	Location (n)	Treatment	Median PFS	Best response	Toxicity grade ≥3
Van Essen et al. (20)	33	Bronchial (3), gastric (1), rectal (1), midgut (15), pancreatic (8), unknown origin (5)	2 cycles of 7.4 GBq ^177^Lu-DOTATATE	17 months	PR: 6 (18.2%) MR: 2 (6.1%) SD: 8 (24.2%) PD: 17 (51.5%) DCR: 16 (48.5%)	Hematologic: n=5
Sabet et al. (21)	33	Pancreatic (14), foregut (3), midgut (6), hindgut (3), other (7)	2-4 cycles. Mean administered activity during re-treatment: 17.7 GBq ^177^Lu-DOTATATE	13 months	CR: 1 (3%) PR: 6 (18.2%) MR: 1 (3%) SD: 14 (42.4%) PD: 11 (33.3%) DCR: 22 (66.6%)	Hematologic: n=7
Severi et al. (22)	26	Pancreatic (17), Ileum (5), appendix (1), colon (1), rectum (1), unknown origin (1)	2-5 cycles. Median activity for re-treatment: 16.5 GBq ^177^Lu-DOTATATE	22 months	CR: 1 (3.8%) PR: 1 (3.8%) SD: 20 (76.9%) PD: 4 (15.4%) DCR: 22 (84.6%)	Renal: n=1. Hematologic: n=1
Yordanova et al. (23)	15	Foregut (8), midgut (3), renal (1), unknown origin (3)	3-6 cycles. Median cumulative activity: 63.9 GBq ^177^Lu-DOTATATE	18.9 months	NA	Hematologic: n=2
Vaughan et al. (24)	47 Re-retreatment: 44	Midgut (21), pancreatic (15), hindgut (2), lung (3), unknown (2), other (2)	^90^Y-Dotatoc: 29 patients, 177Lu-DOTATATE: 18 patients.	17.5 months	PR: 10 (21.27%) SD: 37 (78.72%) DCR: 47 (100%) Re-retreatment: PR: 7 (15.9%) SD: 26 (59.1%) PD: 11 (25%) DCR: 33 (75%)	Renal: n=1 Hematologic: n=2 Myelodysplastic syndrome: n=1
Van der Zwan et al. (25)	168 Re-retreatment: 13	Bronchial (13), pancreatic (53), midgut (54)	Re-treatment: 2 cycles (median cumulative dose: 44.7 GBq), re-retreatment: 2 cycles (median cumulative dose: 59.7 GBq)	14.6 months (14.2 months from re-retreatment)	PR: 26 (15.5%) SD: 100 (59.5%) PD: 33 (19,6%) DCR: 126 (75%) Re-retreatment: PR: 5 (38,5%) SD: 7 (53,8%) PD: 1 (7,7%) DCR: 12 (92,3%)	Hematologic: n=14 Myelodysplastic syndrome: n=2 Acute myeloid leukemia: n=2
Rudisile et al. (26)	35	Midgut (23), lungs (5), unknown primary (4), rectal (1), gastric (1), paraganglioma (1)	1-4 cycles. Median cumulative activity 44 GBq ^177^Lu-DOTATATE	6 months	PR: 1 (3,1%) SD: 26 (81.3%) PD: 5 (15.6%) DCR: 27 (77.1%)	Hematologic: n=1

PR, partial response; MR, minor response; SD, stable disease; PD, progression of disease; DCR, disease control rate; R-PRRT, re-treatment with peptide receptor radionuclide therapy; RR-PRRT, re-retreatment with PRRT.

### Efficacy

Two meta-analysis have been published lately with the aim to evaluate the efficacy and safety of salvage PRRT by Strosberg et al. and Kim et al. (27, 28). These analyses included thirteen and nine articles respectively. Strosberg et al. found a PFS of 12.52 months and an OS of 26.78 months with a disease control rate (DCR) of 71%. Kim et al. reported a PFS of 14.1 months and an OS of 26.8 months and a DCR of 76.9%. However, these meta-analyses present several limitations. These articles found an important heterogeneity between studies, with different doses of salvage PRRT. In addition, the studies also included a small number of patients and presented different criteria regarding toxicity, evaluation of response and outcomes reported.

Van der Zwan et al. published the largest cohort of patients who underwent re-treatment with PRRT with only ^177^Lu-DOTATATE. 181 patients with bronchial or GEP-NET were included in the analysis after receiving salvage therapy with PRRT. Objective response and stable disease were observed in 26 patients (15.5%) and 100 patients (59.5%), respectively. The median PFS was 14.6 months (95% CI 12.4–16.9). In addition, a control group consisting of patients not undergoing salvage therapy, but in principle qualifying for it, was established for estimating the potential increase in OS. Patients re-treated with ^177^Lu-DOTATATE had a significantly longer OS than control patients (p <0.01). In fact, in this series some patients were re-treated twice if the patient had reached disease control after the first salvage PRRT. They found 38.5% of partial responses, 53.8% of stable diseases and a median PFS of 14.2 months. In this subgroup the combined OS after the 3 PRRT treatments was 80.8 months (95% CI 66.0–95.6). The study concluded that ^177^Lu-DOTATATE re-treatment is a suitable option in patients with previous response to PRRT (25).

Other studies have also shown these promising results. The median PFS reported range from 6 to 22 months (22, 26). The short PFS observed by Rudisile et al. compared to other studies could be explained by the late sequence of salvage PRRT, with many intermediate therapies. The DCR was over 50% in the majority of studies, showing more than 80% in the series reported by Severi and Rudisile (22, 26). Yordanova et al. also published overall survival data, achieving 85.6 months against 69.7 months in patients who received only a baseline therapy with PRRT in the same department and time (23).

As expected, efficacy of salvage PRRT is worse than in NETTER-1, with PRRT as first treatment after octreotide analogues (15). There are some reasons, as a lower number of cycles, or the fact that patients have a more advanced disease and worse performance status.

The heterogeneous population included is one of the motives for the variable outcomes. First of all, these studies included NETs from different primary tumor locations: all of them included gastroenteropancreatic and unknown origin tumours, but some series also included bronchial tumors (20, 26), and other study included even paragangliomes or medullary thyroid carcinomas (24).

These results support the strategy of PRRT re-treatment in patients with NETs. However, there are several limitations in the interpretation of these data due to the heterogeneity between studies and the small number of patients included in these studies.

### Toxicity

Hematological and renal toxicity are the main side effects and dose limiting factors for PRRT. However, the safety profiles of ^177^Lu-DOTATATE and ^90^Y-DOTATATE are different, particularly renal toxicity is more often reported with ^90^Y-DOTATATE.

Previously mentioned meta-analysis found a similar toxicity profile between salvage PRRT and initial PRRT. Strosberg et al. described a 5% of grade 3/4 adverse events and 0% of renal toxicity, and Kim et al. found a 10.8% of hematologic toxicity and 0.7% of renal toxicity (27, 28).

In the series, the most common toxicity observed was hematologic with grade 3/4 in 4% to 7% of patients, similar to results with ^177^Lu-DOTATATE in NETTER-1 (15). In addition, 1% of patients developed late toxicity as acute myeloid leukemia or myelodysplastic syndrome, showed in a study which evaluates not only re-treatment, but also a second re-treatment (25). The data of Sabet et al. stands out because they showed a hematologic toxicity grade 3 or more in 21% of patients, without higher accumulated dose (21) (the mean accumulate activity was 44.3 GBq, while the rest of the studies had a similar range, reaching 63.8 GBq in one series (23)). In a lower number of patients, renal toxicity appears, but it is exclusively observed with ^90^Y-DOTATATE (up to 4% grade 3/4) (22, 24). Although personalized dosimetry was not used routinely in these studies, the possibility of including this measurement could guide the treatment planning and control the absorbed dose to vulnerable organs (kidneys and bone marrow).

### Patient Selection

This is the most important point in PRRT re-treatment. The selection of patients suitable for this strategy will focus our effort to optimize the benefit of PRRT. There are some considerations in which there is more agreement. For example, patients must have a significant clinical benefit after treatment with a previous PRRT to be considered eligible for re-treatment. The controversy is how to measure this benefit. In this way, there are differences in the duration of the clinical benefit: some series request at least 12 months after the last cycle of previous PRRT (20, 22, 24), whereas there is another study that demands at least 18 months (25). The PFS after the first PRRT treatment has been identified as the main factor to predict more durable benefit to salvage ^177^Lu-DOTATATE (20, 21). Consequently, treatment outcome was less favorable in patients with a short PFS after the first PRRT treatment.

Although there are no other factors clearly associated with response to PRRT re-treatment, some of them have been described as potential predictive markers. However, one of the limitations of this review is that characteristics of patients that receive PRRT re-treatment were not consistently reported across the studies.

The tumour uptake in the somatostatin receptor scintigraphy and ^68^Ga-DOTATATE PET has a known predictive role to predict response of PRRT in NETs (29, 30). Sufficient radiotracer uptake on SSTR imaging was an indication for PRRT re-treatment in the majority of studies. Van Essen et al. described a higher tumour uptake in patients that received a second treatment with ^177^Lu-DOTATATE compared with a group of patients treated with the regular therapy (20). However, these findings contrast with other data reported (22), in which the degree of scintigraphy uptake at baseline did not correlate with PFS and OS.

Severi et al. also described a relation between survival after PRRT re-treatment and tumour burden. Patients with an extensive disease, especially those with liver metastases had a shorter OS (22). Despite these data, their potential predictive role is unclear because tumour burden disease and liver involvement are common prognostic factors in oncologic patients.

Tumour dedifferentiation is associated with somatostatin receptor expression. Poorly-differentiated tumors have a lower expression and tumour uptake in the somatostatin receptor scintigraphy with a more aggressive behavior. In this way some authors have described a worse response with PRRT in these patients (20).

The presence of a functional tumor could guide our treatment decision. The current evidence about the efficacy of PRRT regarding control of carcinoid syndrome (CS), showed a symptoms reduction of up to 87,8% (31, 32). The appearance or persistence of an uncontrolled CS could be another factor to consider when evaluating which patients could benefit from the re-treatment.

### Schedule of Salvage PRRT

There are also differences in the treatments administered. In this way, different radionuclides have been used as previous treatment or as re-treatment. Severi et al. published a study analyzing 26 patients who received ^177^Lu-DOTATATE after progression to ^90^Y-Dotatoc. Median PFS is 22 months with a control disease rate of 84.6% (22). The series reported by Van der Zwan et al. included 181 treated with ^177^Lu-DOTATATE as initial and salvage PRRT, with a median PFS of 14.6 months (25). Finally, Vaughan et al analyzed retrospectively 47 patients, 45 of them were treated with ^90^Yttrium (^90^Y)-DOTATATE and 2 with ^177^Lu-DOTATATE as initial treatment. The re-treatment was with ^90^Y-DOTATATE in 29 patients and with ^177^Lu-DOTATATE in 18 patients. Median PFS was 17.5 months, and no statistically significant differences between both drugs were observed (24).

In addition, there is no consensus about the number or dose of cycles administered: from the fixed two additional cycles in one study (20), up to the six cycles reached in the retrospective study of Yordanova et al. (23). Despite the majority of the studies use a similar per cycle dose of 7.4 GBq, the study of Severi et al. selected a lower dose of 3.7 GBq (22). Furthermore, there is also diversity in the time of salvage therapy. Some studies included patients who receive salvage with PRRT as the first treatment after progression to previous PRRT, but others include extensively pretreated patients with several intermediate treatments (20, 23, 26).

### Re-Treatment PRRT in the Guidelines

Treatment guidelines of NETs include the option of salvage PRRT. The Joint International Atomic Energy Agency (IAEA), the European Association of Nuclear Medicine (EANM), and the Society of Nuclear Medicine and Molecular Imaging (SNMMI) accept PRRT re-treatment in patients with previous response, with the same inclusion criteria used in the initial treatment and paying special attention to accumulated doses in bone marrow and kidney (33). The North American Neuroendocrine Tumour Society (NANETS)/SNMMI Consensus Statement on Patient Selection and Appropriate Use of ^177^Lu-DOTATATE PRRT remark the efficacy and the acceptable toxicity demonstrated by the studies which have evaluated re-treatment (34). In contrast, the latest guidelines of NCCN or ESMO do not propose salvage with PRRT as an option (35, 36).

## Conclusion

In conclusion, our patient is a good example of re-treatment with PRRT, due to its initial response to ^177^Lu-DOTATATE, which lasted more than 3 years. In that moment, two additional cycles of PRRT were administered, reaching again partial response without significant toxicity. ^177^Lu-DOTATATE is an effective therapy in NETs with an excellent safety profile. There is evidence that salvage therapy following progression to PRRT after a long response is an option in these patients, with high disease control rates and acceptable safety profile. Nevertheless, large prospective randomized studies are needed to confirm these findings.

## Data Availability Statement

The raw data supporting the conclusions of this article will be made available by the authors, without undue reservation.

## Ethics Statement

Written informed consent was obtained from the individual(s) for the publication of any potentially identifiable images or data included in this article.

## Author Contributions

EV wrote the manuscript with support from PG, AM, and AS provided the images. All authors provided critical feedback and helped shape the analysis and manuscript, and participated in the management and follow-up of the patient. All authors contributed to the article and approved the submitted version.

## Funding

This research did not receive any specific grant from any funding agency in the public, commercial or not-for-profit sector.

## Conflict of Interest

EG has served as advisor and delivered lectures for Pfizer, Bristol-Myers Squibb, Ipsen, Roche, Eisai, Eusa Pharma, MerckSharp&Dohme, Sanofi-Genzyme, Adacap, Novartis, PierreFabre, Lexicon and Celgene. PG has served as advisor and delivered lectures for Pfizer, Bristol-Myers Squibb, Ipsen, Roche, Eisai, Sanofi-Genzyme, Adacap, Novartis.

The remaining authors declare that the research was conducted in the absence of any commercial or financial relationships that could be construed as a potential conflict of interest.
